# Increased Levels of Adipocyte and Epidermal Fatty Acid-Binding Proteins in Acute Lymphoblastic Leukemia Survivors

**DOI:** 10.3390/jcm10081567

**Published:** 2021-04-08

**Authors:** Katarzyna Konończuk, Eryk Latoch, Beata Żelazowska-Rutkowska, Maryna Krawczuk-Rybak, Katarzyna Muszyńska-Rosłan

**Affiliations:** 1 Department of Pediatric Oncology and Hematology, Medical University of Bialystok, 15-274 Bialystok, Poland; rybak@umwb.edu.pl (M.K.-R.); kmroslan@post.pl (K.M.-R.); 2 Department of Pediatric Laboratory Diagnostic, Medical University of Bialystok, 15-274 Bialystok, Poland; zelazowskab@wp.pl

**Keywords:** FABP, A-FABP, E-FABP, overweight, obesity, children, metabolic syndrome, ALL, acute lymphoblastic leukemia, CCS, childhood cancer survivors

## Abstract

Childhood cancer survivors are highly exposed to the development of side effects after many years of cessation of anticancer treatment, including altered lipid metabolism that may result in an increased risk of overweight and metabolic syndrome. Adipocyte (A-FABP) and epidermal (E-FABP) fatty acid-binding proteins are expressed in adipocytes and are assumed to play an important role in the development of lipid disturbances leading to the onset of metabolic syndrome. The aim of this study was to investigate the association between serum A-FABP and E-FABP levels, overweight, and components of the metabolic syndrome in acute lymphoblastic leukemia survivors. Sixty-two acute lymphoblastic leukemia (ALL) survivors (34 females) were included in the study. The mean age at the time of the study was 12.41 ± 4.98 years (range 4.71–23.43). Serum levels of A-FABP and E-FABP were analyzed using a commercially available ELISA kit. The ALL survivors presented statistically higher A-FABP levels in comparison with the healthy controls (25.57 ± 14.46 vs. 15.13 ± 7.61 ng/mL, *p* < 0.001). The subjects with body mass index (BMI) above the normal range (18 overweight, 10 obese) had a greater level of A-FABP compared to the ALL group with normal BMI (32.02 ± 17.10 vs. 20.33 ± 9.24 ng/mL, *p* = 0.006). Of all participants, 53.23% had at least one risk factor of metabolic syndrome; in this group, only the A-FABP level showed a statistically significant difference compared to the healthy control group (30.63 ± 15.91 vs. 15.13 ± 7.61 ng/mL, *p* < 0.001). The subjects with two or more metabolic risk factors (16.13%) presented higher levels of both A-FABP (33.62 ± 17.16 vs. 15.13 ± 7.61 ng/mL, *p* = 0.001) and E-FABP (13.37 ± 3.62 vs. 10.12 ± 3.21 ng/mL, *p* = 0.021) compared to the controls. Univariable regression models showed significant associations between BMI and systolic blood pressure with the A-FABP level (coeff. 1.02 and 13.74, respectively; *p* < 0.05). In contrast, the E-FABP level was only affected by BMI (coeff. 0.48; *p* < 0.01). The findings reported herein suggest that the increased levels of A-FABP and E-FABP may be involved in the pathogenesis of overweight and the onset of metabolic syndrome in acute lymphoblastic leukemia. However, further longitudinal, prospective studies of fatty acid-binding proteins and their potential role in the pathogenesis of obesity and metabolic syndrome in ALL survivors remain to be performed.

## 1. Introduction

Acute lymphoblastic leukemia (ALL) constitutes over 25% of all childhood cancers, which qualifies it to be the most common childhood malignancy. Due to significant advances in the diagnosis and treatment of ALL in recent years, the improvement in survival rate up to 90% has been reached depending on the risk group [1,2,3]. As a result, the number of survivors increases dramatically with the improvement of treatment results. On the other hand, many studies indicate that this population is exposed to numerous treatment-related complications in later life [4,5,6]. Therefore, there has recently been a growing interest in the detection and prevention of late sequelae of anticancer treatment among this population. 

Over the past two decades, multiple reports have been published that indicate childhood cancer survivors (CCS) are at risk of premature aging and develop many diseases earlier than the general population [7]. The most common complications include diseases of civilization such as obesity, diabetes mellitus, metabolic syndrome, heart diseases, osteoporosis or second cancers [8,9]. To a large extent, this may be related to the treatment applied in this group of patients; however, the underlying processes are multifactorial and still not fully explained. To date, cranial radiation has been noted to play an important role in the development of metabolic syndrome (MetS) and insulin resistance (IR) [10]. Other adverse factors include the use of anticancer agents, glucocorticosteroids, as well as decreased physical activity and poor eating habits, which further result in the difficulty of reversing unhealthy habits among survivors many years after the treatment.

All of these factors boost the risk of cardiovascular complications and overweight in ALL survivors than their peers [11]. Moreover, the available studies have demonstrated that anticancer treatment may alter lipid metabolism; however, prognostic markers that can be used in clinical practice are limited [12]. 

Fatty acid-binding proteins (FABPs) are a family of proteins involved in the regulation of lipid and glucose metabolism. As lipid chaperones, they can bind to saturated and unsaturated long-chain fatty acids, which is important to coordinate lipid migration to different cellular compartments. FABP-4 is also known as adipocyte-FABP (A-FABP), which is produced in adipocytes. Some authors indicate that overweight individuals have a higher concentration of A-FABP in the bloodstream, which is strongly correlated with body fat mass [13]. 

Epidermal FABP (E-FABP) is released mostly by epidermal cells of the skin as well as by adipocytes and other cells and tissues, i.e., macrophages, lens, lung, and brain [14]. Both of these FABPs also play a significant role in the development of insulin resistance and atherosclerosis [15,16]. 

This study aimed at investigating the association between the A-FABP and the E-FABP levels, overweight, and components of the metabolic syndrome in acute lymphoblastic leukemia survivors. 

## 2. Materials and Methods

The study included 62 childhood cancer survivors (28 male and 34 female) of the Department of Pediatric Oncology and Hematology of the Medical University of Bialystok. All the patients were treated for acute lymphoblastic leukemia. The mean age at the time of the study was 12.41 ± 4.98 years (range 4.71–23.43). The characteristics of the patients are presented in Table 1. International protocols (The International Berlin-Frankfurt-Münster Group—I-BFM) approved by Polish Pediatrics Leukemia and Lymphoma Group were used in the treatment. Hematological stem cell transplantation (HSCT) was performed in 6 participants. All the patients were in complete continuous remission. Written informed consent was obtained from all the subjects. The control group consisted of 25 healthy peers (10 female) born on time, with normal body weight and BMI during the examination and did not take any medication, who were offspring of the department’s employees. The study was approved by the Ethics Committee of the Medical University of Bialystok in accordance with the Declaration of Helsinki (permission number: R-I-002/463/2016).

Data on age, sex, type of diagnosis, and treatment were collected from the medical records. All the patients underwent a clinical examination and anthropometric measurements during the follow-up visit. A Martin anthropometer was used to measure height and a digital scale to weigh (Seca, Germany). Body mass index (BMI) was calculated as weight in kilograms divided by height in squared meters (kg/m^2^). The subjects were divided into overweight and obesity groups and a group with BMI in a normal range based on the OLA/OLAF growth charts of BMI for age and sex. Overweight was defined as BMI values +1 SD, while obesity was defined as +2 SD [17,18]. The waist-to-height ratio (WHtR) was calculated by dividing waist circumference by height, assuming abdominal obesity as WHtR ≥ 0.5 [19]. Blood pressure was measured using a standardized sphygmomanometer (performed three times at 1–2 min intervals); before the measurement, the participant rested peacefully for 5 min. Hypertension (HT) was defined as a mean systolic blood pressure (SBP) and/or diastolic blood pressure (DBP) level ≥  95th percentile adjusted for age, sex, and height [20].

The components of metabolic syndrome in children under the age of 16 were defined by the International Diabetes Federation (IDF) recommendations as waist circumference (WC) ≥ 90th centile, triglycerides ≥ 150 mg/dL (≥1.7 mmol/L), high-density lipoprotein (HDL) cholesterol < 40 mg/dL (<1.03 mmol/L), blood pressure ≥ 130/85 mmHg, and fasting glucose ≥ 100 mg/dL (≥5.6 mmol/L). Among participants aged 16 and older, MetS was defined by the IDF adult criteria as WC ≥ 94 cm for men and WC ≥ 80 cm for women, triglycerides ≥ 150 mg/dL (≥1.7 mmol/L), HDL cholesterol < 40 mg/dL (<1.03 mmol/L) for men and < 50 mg/dL (<1.29 mmol/L) for women, blood pressure ≥ 130/85 mmHg, fasting glucose ≥ 100 mg/dL (≥5.6 mmol/L) [21]. All the subjects had an abdominal ultrasound performed by a qualified radiologist to assess the occurrence of liver steatosis. Shortening fraction (SF) and ejection fraction (EF) evaluation in echocardiography were measured by a pediatric cardiology specialist.

All the laboratory tests were performed following an eight-hour overnight fast. Blood samples were stored at −80 °C. Serum levels of A-FABP and E-FABP were analyzed using a commercially available ELISA kit (BioVendor Laboratorni Medicina a.s., Brno, Czech Republic). Additionally, concentrations of biochemical parameters such as alanine aminotransferase (ALT), high-density lipoprotein (HDL), triglycerides (TG), and glucose were measured by using the enzymatic colorimetric methods.

Statistical analysis was performed with STATA v. 12.1 (StatCorp, College Station, Texas, USA). The Shapiro–Wilk test was used to examine normal distribution. The data were expressed as means ± standard deviation (SD), or median (Me) and interquartile range (IQR) when appropriate. The Mann–Whitney U test was applied to compare independent variables without normal distribution. The correlation analysis between parameters was evaluated with Spearman’s rank correlation coefficient. The receiver operating characteristic (ROC) curve was used to establish the diagnostic values of the fatty acid-binding proteins and the optimum cut-off points. Multivariate regression models were used to examine the association between FABPs and the independent variables, which potentially might affect their level. The level of statistical significance was defined as *p* < 0.05.

## 3. Results

The characteristics of the included childhood cancer survivors (CCS) are shown in Table 1. The mean age at the time of diagnosis was 5.03 ± 3.37 years, and the mean time from treatment cessation to follow-up was 6.61 ± 4.59 years. The anthropometric analyses and biochemical parameters measured in the study according to sex are presented in Table 2. The age and sex of the study group did not differ from the control group. 

**Figure 1:**
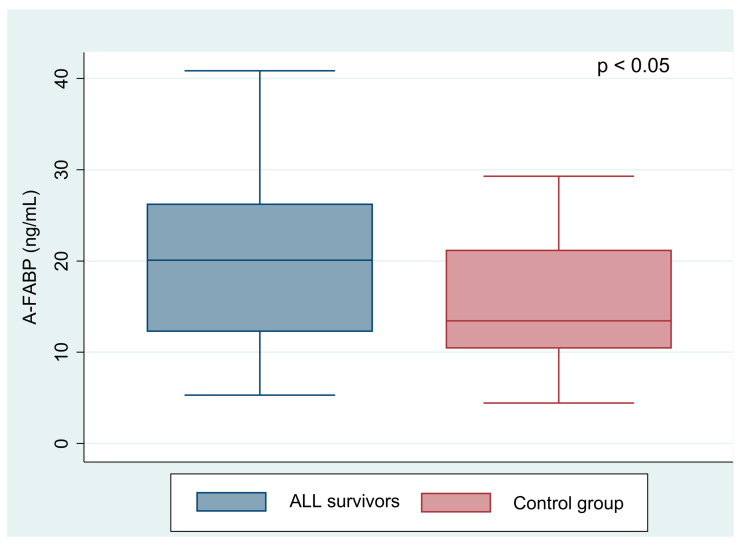
Comparison of the A-FABP level (adipocyte fatty acid-binding protein) between acute lymphoblastic leukemia survivors with normal BMI and the control group.

**Figure 2:**
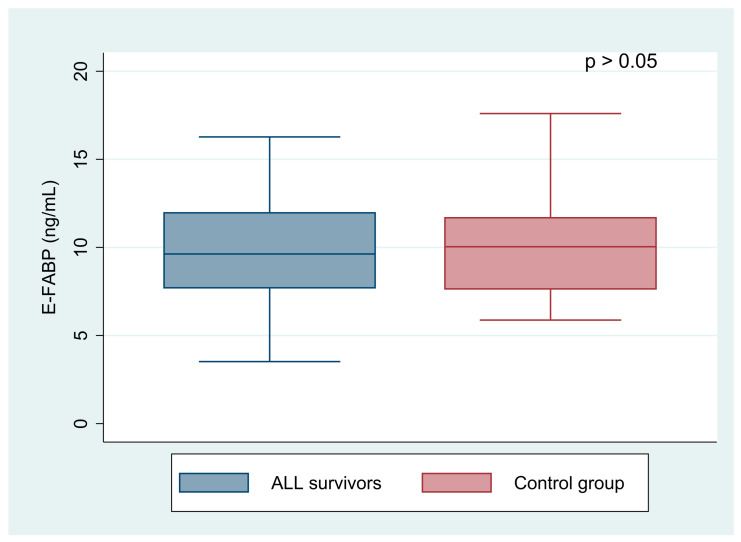
Comparison of the E-FABP level (epidermal fatty acid-binding protein) between acute lymphoblastic leukemia survivors with normal BMI and the control group.

ALL survivors had statistically higher A-FABP level than the healthy controls (25.57 ± 14.46 vs. 15.13 ± 7.61 ng/mL, *p* < 0.001). In contrast, the E-FABP level showed no differences in the groups concerned (12.07 ± 7.37 vs. 10.12 ± 3.21 ng/mL, *p* = 0.325). Comparisons of A-FABP and E-FABP levels in acute lymphoblastic leukemia patients with normal BMI with controls are shown in Figure 1 and Figure 2. The level of E-FABP was statistically significantly higher in the examined females than in males (13.59 ± 8.80 vs. 10.33 ± 4.87 ng/mL, *p* = 0.023). However, A-FABP did not reveal any differences according to sex (26.69 ± 14.69 vs. 24.05 ± 14.32 ng/mL, *p* = 0.349).

Twenty-eight of the study group participants (45%) were overweight (*n* = 18) or obese (*n* = 10). All of them had a significantly higher level of A-FABP compared to the ALL group with normal BMI (32.02 ± 17.10 vs. 20.33 ± 9.24 ng/mL, *p* = 0.006). No differences in concentrations of E-FABP (13.85 ± 8.97 vs. 10.61 ± 5.46 ng/mL, *p* = 0.055), TG (107.95 ± 47.88 vs. 87.41 ± 45.62 mg/dL, *p* = 0.078), and ALT (32.38 ± 36.61 vs. 19.79 ± 16.92 U/L, *p* = 0.053) were found in these subjects (Table 3).

In the analysis by gender, female subjects with normal BMI showed a higher concentration of A-FABP compared to the control group (22.74 ± 8.28 vs. 11.87 ± 6.77 ng/mL, *p* < 0.001), whereas the male subjects with normal BMI did not reveal significantly statistical differences compared to the control group (A-FABP: 16.80 ± 9.75 vs. 17.30 ± 7.56 ng/mL, *p* = 0.717). Additionally, the higher levels of both A-FABP and E-FABP were found in females with high BMI in comparison to the control group (E-FABP: 16.07 ± 11.85 vs. 11.87 ± 6.77 ng/mL, *p* = 0.007; A-FABP: 34.32 ± 20.23 vs. 11.87 ± 6.77 ng/mL, *p* = 0.001). We observed a similar dependence in overweight male subjects, but it concerned only the A-FABP concentration (30.33 ± 14.93 vs. 17.30 ± 7.56 ng/mL, *p* = 0.006). 

**Figure 3:**
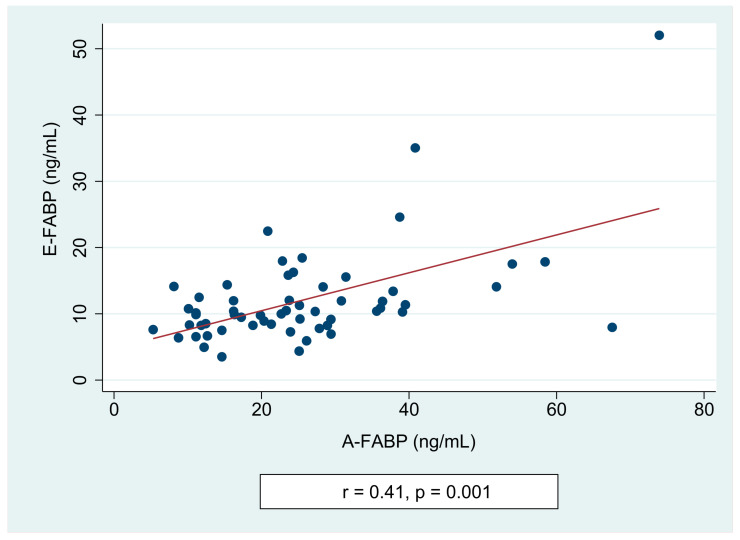
Spearman correlation of A-FABP (adipocyte fatty acid-binding protein) and E-FABP (epidermal fatty acid-binding protein) in childhood acute lymphoblastic leukemia survivors.

We did not notice any differences in the FABPs levels in patients with and without cranial irradiation (E-FABP: 12.25 ± 7.77 vs. 11.05 ± 4.49 ng/mL, *p* = 1.000; A-FABP: 25.98 ± 15.00 vs. 23.03 ± 10.92 ng/mL, *p* = 0.748), respectively. We found a positive correlation between the E-FABP and the A-FABP concentrations in ALL survivors (r = 0.41, *p* = 0.001) (Figure 3).

Moreover, we observed moderate correlations between FABPs and anthropometric parameters such as BMI (E-FABP: *r* = 0.42, *p* = 0.001; A-FABP: *r* = 0.43, *p* = 0.001), WHtR (E-FABP: *r* = 0.35, *p* = 0.007; A-FABP: *r* = 0.37, *p* = 0.004), WC (E-FABP: *r* = 0.40, *p* = 0.002; A-FABP: *r* = 0.40, *p* = 0.002), and fatty acid-binding proteins. In addition, a positive correlation between high systolic blood pressure and the analyzed biomarkers were found (E-FABP: *r* = 0.29, *p* = 0.023; A-FABP: *r* = 0.35, *p* = 0.007), while diastolic blood pressure was only positively associated with A-FABP (*r* = 0.32, *p* = 0.015); however, the strength of relationships was weak (0.3 < *r* < 0.5) or very weak (*r* < 0.3). We did not find any correlation between FABPs and shortening fraction (SF) or ejection fraction (EF). The multiple regression models were developed regarding each variable that might affect the level of FABPs. The analysis included potential confounding variables listed in Table 1 and Table 2. The best models (with the highest coefficient of determination) of the relationship between FABPs and independent variables are presented in Table 4. The first model showed that BMI and SBP significantly affected A-FABP level (coeff. 1.02 and 13.74, respectively; *p* < 0.05), while the second one revealed that only BMI significantly influenced the E-FABP level (coeff. 0.48; *p* = 0.005).

**Figure 4:**
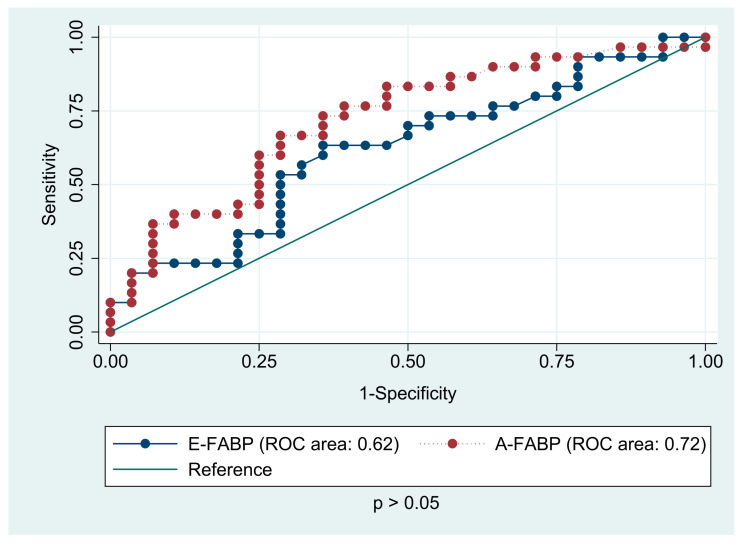
The receiver operating characteristic (ROC) analysis for prediction of the presence of components of metabolic syndrome based on the serum levels of A-FABP (adipocyte fatty acid-binding protein) and E-FABP (epidermal fatty acid-binding protein) in childhood acute lymphoblastic leukemia survivors.

**Figure 5:**
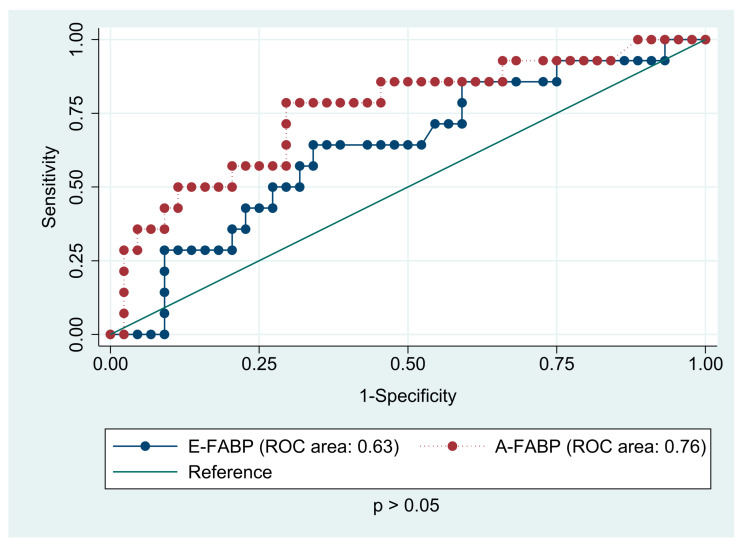
The receiver operating characteristic (ROC) analysis for prediction of overweight/obesity based on the serum levels of A-FABP (adipocyte fatty acid-binding protein) and E-FABP (epidermal fatty acid-binding protein) in childhood acute lymphoblastic leukemia survivors.

The receiver operating characteristic analyses (Figure 4) for predicting the presence of any components of metabolic syndrome based on the serum levels of A-FABP and E-FABP in the study group were conducted. Adipocyte FABP (AUC 0.72) was found to be a better predictor then E-FABP (AUC 0.62), yet no statistical differences were observed between the two areas under the curve (*p* > 0.05). The same analyses for prediction of overweight and obesity (Figure 5) showed similar results (AUC 0.76 for A-FABP and 0.63 for E-FABP, *p* < 0.05). Interestingly, the examined proteins turned out to be predictors of higher blood pressure in CCS. The AUC for A-FABP was 0.85 for SBP and 0.69 for DBP, while E-FABP showed diagnostic profile describing the AUC of 0.70. There was no diagnostic value of FABPs in hepatic steatosis detected by ultrasound. 

In further analysis, we checked the relationship between the FABPs and the anthropometric measurements depending on the time elapsed since the end of treatment. The subjects over 5 years after cessation of therapy presented greater A-FABP (27.85 ± 14.22 vs. 22.10 ± 14.44 ng/mL, *p* = 0.045) and BMI (22.76 ± 4.89 vs. 20.09 ± 5.20 kg/m^2^, *p* = 0.032) than participants with a shorter time after completion of treatment. We found no differences in E-FABP concentration in these patient subgroups.

Among all participants, at least one risk factor of metabolic syndrome was noticed in 53.23%. In this group, a higher concentration of A-FABP compered to subjects without MetS features was found (30.63 ± 15.91 vs. 20.14 ± 10.52 ng/mL, *p* = 0.003). Interestingly, the subset with two or more metabolic risk factors showed a significant difference in the concentration of both proteins (A-FABP: 33.62 ± 17.16 vs. 20.27 ± 10.35 ng/mL, *p* = 0.018; E-FABP: 13.37 ± 3.62 vs. 10.19 ± 4.02 ng/mL, *p* = 0.026). When compared to the control group, the patients with any metabolic derangements had higher levels of A-FABP (30.63 ± 15.91 vs. 15.13 ± 7.61 ng/mL, *p* < 0.001) but not E-FABP (13.73 ± 9.25 vs. 10.12 ± 3.21 ng/mL, *p* = 0.090). The participants with two or more metabolic risk factors (16.13%) presented significantly higher levels of A-FABP (33.62 ± 17.16 vs. 15.13 ± 7.61 ng/mL, *p* = 0.001) and E-FABP (13.37 ± 3.62 vs. 10.12 ± 3.21 ng/mL, *p* = 0.021) than the control group (Table 5). Due to a small number of patients, we did not analyze CCS with three or four metabolic risk factors separately.

## 4. Discussion

The association between obesity-related risk factors and anticancer treatment during childhood has been established [22]. However, metabolic pathways leading to the development of obesity and its complications have not been fully elucidated in this group of patients. In this cross-sectional study, we sought to evaluate whether serum levels of A-FABP and E-FABP are elevated in acute lymphoblastic leukemia survivors and to assess their relationship with overweight and features of metabolic syndrome. 

The studies on the importance of fatty acid-binding proteins in various diseases have been conducted [14,15]. The increased FABPs levels have been previously reported among the patients with obesity, hyperglycemia, insulin resistance, type 2 diabetes mellitus, hypertension, left ventricular diastolic dysfunction, atherosclerosis, and heart failure [13,23,24,25,26,27,28,29,30]. Moreover, in a 5-year prospective study, A-FABP has been noted to be a significant predictor of the occurrence of metabolic syndrome regardless of adiposity and insulin resistance [24]. Other studies showed that a higher level of E-FABP positively correlated with the components of MetS, yet it was not as strongly correlated as A-FABP [15,16]. Few studies on A-FABP levels have been conducted in children. Some of them indicate its higher level in overweight children and a possible association with the development of MetS [31,32].

In this study, elevated levels of A-FABP, but not E-FABP, were found among ALL survivors compared to healthy peers. These results support the hypothesis that this particular group of subjects may be susceptible to altered lipid metabolism due to anticancer treatment used in childhood. It was also shown that overweight subjects had higher levels of A-FABP compared to normal-weight subjects, which may indicate the presence of greater metabolic disturbances among overweight ALL survivors. Since A-FABP is expressed mainly in adipocytes, it may be a better predictor of lipid metabolism changes than E-FABP, which is also secreted by other tissues less involved in fat metabolism. 

This study also showed that overweight females had higher concentrations of both A-FABP and E-FABP than the female control group. In contrast, overweight males had only higher levels of A-FABP compared to healthy male controls. The underlying mechanism of the gender difference in serum A-FABP concentration has not been fully elucidated. However, Hu et al. [33] showed a negative correlation between the androgen and A-FABP levels in men, but a positive correlation in women. An additional factor contributing to this difference may be the natural variation in body fat distribution according to gender [15].

Many large epidemiologic studies have demonstrated that pediatric ALL survivors treated with cranial radiotherapy are particularly vulnerable to metabolic disturbances and unhealthy weight gain regardless of sex or weight status before the treatment [22,34,35,36]. In the present study, we found no differences according to the radiotherapy used. However, the number of individuals with a cranial radiotherapy (CRT) history was too small to conduct a reliable analysis.

The presence of one or more metabolic risk components among ALL survivors accounted for 53.23%. Data from the literature indicate that the population of CCS is more likely to develop metabolic syndrome, yet not all risk factors have been identified [35]. Smith et al. [37] reported that over 30% of adult CCS with the mean follow-up time after treatment discontinuation 25.6 years suffered from MetS, and the most common component in men was high blood pressure, while women had more often low HDL. According to other studies, the incidence of MetS was associated with male sex, age, and BMI at diagnosis. However, there are still inconsistent data in this field, with some papers suggesting that it is the female gender that increases the risk of developing MetS [38,39]. In our study, only one patient met all criteria of MetS, but roughly half of the survivors (children and young adults) met at least one criterion, which may indicate the early onset of the MetS. Xu et al. [13] have reported that A-FABP concentration is associated with obesity and the higher number of metabolic syndrome components in the general population in both genders, which is in accordance with our results. Only an increased A-FABP level in a subset of subjects who met at least one MetS criterion was identified in the current study. On the other hand, those who met at least two criteria had higher concentrations of both A-FABP and E-FABP than the control group or participants who did not meet any criteria. Our study supports the findings of previous research studies that CCS are more likely to develop the components of MetS. However, further studies on a larger group of patients are needed to determine the suitability of fatty acid-binding proteins for distinguishing a subset of CCS at high risk of developing MetS. 

Furthermore, Levy et al. [40] described that ALL survivors were significantly at a higher risk of having pre-hypertension and hypertension than healthy counterparts. In our study, we observed a positive correlation between A-FABP and E-FABP and systolic blood pressure, although they were within the normal range. This may be a cause of hypertension in the future, but further studies are needed. All of these individuals should have their blood pressure checked regularly. 

Interestingly, Oeffinger et al. [41] reported that ALL survivors were significantly more predisposed to develop insulin resistance even when compared to a control group 10 years older. Meachan et al. [42] showed that CCS were more likely to take medications for diabetes than controls. Our previous study examining obesity and insulin resistance among CCS supports these findings [43].

Some studies showed that obese patients diagnosed with cancer had higher levels of A-FABP than normal weight patients. In addition, these patients exhibit greater cancer progression [44,45]. Thus, the potential association between A-FABP and obesity in ALL survivors may already be due to the primary alteration in lipid metabolism. It is difficult to unequivocally answer the question of whether it is the FABPs that promote the development of ALL-associated MetS or vice versa. A long-term prospective study should be conducted to find the underlying process.

There are several limitations to this study. It was a single-center analysis, with a relatively small number of participants. No analysis of total body fat mass was performed; thus, the relationship between the visceral fat percentage and the levels of analyzed FABPs could not be determined. Furthermore, due to the age of the study population, we did not analyze the insulin resistance; however, this is an important component linked to MetS and should be considered in longitudinal studies. The study could have the added value of comparing the results, with a control group of obese children, which was not considered in this cross-sectional study. This would allow for a better understanding of whether differences in FABPs levels are due to cancer treatment or obesity itself. To conclusively confirm that elevated FABPs in normal weight ALL survivors after treatment promotes obesity later in life, long-term prospective studies would have to be conducted in these patients.

The strengths of our research include the homogenous group of acute lymphoblastic leukemia, relatively long follow-up time, and no ethnic diversity. Moreover, to our best knowledge, this is the first study assessing the role of fatty acid-binding proteins in the development of obesity and metabolic syndrome in acute lymphoblastic leukemia survivors. 

In conclusion, this is the first study that demonstrated that ALL survivors present higher A-FABP levels than the control group. Moreover, the elevated levels of A-FABP and E-FABP were observed in overweight subjects and those with metabolic syndrome features. These findings suggest that the increased levels of fatty acid-binding proteins may be involved in the pathogenesis of overweight and the onset of metabolic syndrome in acute lymphoblastic leukemia survivors. Further longitudinal, prospective analyses of the A-FABP and E-FABP levels in ALL survivors and their potential role as the biomarkers in the pathogenesis of overweight, insulin resistance, and cardiovascular complications remain to be performed.

## Figures and Tables

**Table 1:** Descriptive characteristics of the study group.

	Number (%)	Median (IQR) ^a^
Patients	62 (100%)	
Male	28 (45.2%)	
Female	34 (54.8%)	
Age at diagnosis (years)		4.13 (3.03–6.45)
Age at study (years)		12.39 (8.17–16.08)
Follow-up after treatment (years)		7.05 (2.24–9.07)
Overweight	18 (29%)	
Obese	10 (16%)	
Glucocorticoids:		
Cumulative corticosteroid dose (mg/m^2^) ^c^	62 (100%)	3087 (3087–3087) ^b^
Prednisone (cumulative dose in mg/m^2^)	62 (100%)	1680 (1680–1680) ^b^
Dexamethasone (cumulative dose in mg/m^2^)	62 (100%)	210 (210–210) ^b^
Radiotherapy	9 (14.5%)	
Cranial radiotherapy (CRT) (cumulative dose in Grey)	8 (12.9%)	12 (12–12) ^b^
Total body irradiation (TBI)	2 (3.23%)	12 (12–12) ^b^
No	53 (85.5%)	
HSCT	6 (9.7%)	
Metabolic derangements		
1 Metabolic risk factor	23 (37.1%)	
2 Metabolic risk factors	5 (8.1%)	
3 Metabolic risk factors	4 (6.5%)	
4 Metabolic risk factors	1 (1.6%)	

^a^ Interquartile range (IQR). ^b^ Most patients received the same dosage of anticancer agents according to the treatment protocol; therefore, the first and third quartiles did not differ from the median. ^c^ Calculated as prednisone equivalents.

**Table 2:** Characteristics of acute lymphoblastic leukemia survivors by gender.

	Total	Females	Males	*p* Value
	Median (IQR)	Median (IQR)	Median (IQR)	
	*n* = 62	*n* = 34	*n* = 28	
Age at diagnosis (years)	4.13 (3.03; 6.45)	5.23 (2.91; 7.04)	3.74 (3.30; 5.59)	0.784
Age at study (years)	12.36 (8.17; 16.08)	13.55 (10.13; 16.40)	10.89 (6.51; 14.49)	0.164
Follow-up (years)	7.05 (2.24; 9.07)	7.69 (3.45; 9.36)	5.71 (1.71; 8.68)	0.178
Weight (kg)	47.50 (31.30; 65.00)	49.25 (38.00; 62.70)	44.05 (24.80; 71.35)	0.598
Height (cm)	151.25 (133.50; 162.00)	152.50 (140.00; 160.00)	145.75 (118.75; 166.75)	0.648
BMI (kg/m^2^)	21.17 (17.93; 24.96)	21.41 (18.82; 24.96)	20.99 (17.53; 24.51)	0.817
WC (cm)	72.00 (63.00; 81.00)	72.50 (65.00; 80.00)	71.50 (57.50; 83.00)	1.00
WHtR	0.50 (0.45; 0.54)	0.48 (0.45; 0.53)	0.50 (0.45; 0.55)	0.425
ALT (U/L)	15.00 (12.00; 23.00)	14.00 (12.00; 22.00)	17.00 (13.00; 23.00)	0.513
TG (mg/dL)	91.00 (62.00; 118.00)	84.00 (62.00; 100.00)	98.00 (63.00; 132.00)	0.443
E-FABP (ng/mL)	10.32 (8.26; 14.08)	11.07 (9.43; 15.08)	9.04 (7.12; 12.00)	0.023
A-FABP (ng/mL)	23.69 (14.62; 30.82)	24.71 (16.21; 31.43)	23.09 (11.66; 30.12)	0.349

BMI body mass index, WC waist circumference, WHtR waist-to-height ratio, ALT alanine aminotransferase, TG triglycerides, E-FABP epidermal fatty acid-binding protein, A-FABP adipocyte fatty acid-binding protein, IQR interquartile range.

**Table 3:** Childhood cancer survivors according to different body mass index.

	Overweight/Obese	Normal Weight	*p* Value
	*n* = 28	*n* = 34	
	Median (IQR)	Median (IQR)	
BMI (kg/m^2^)	25.43 (22.36; 28.47)	18.85 (16.18; 21.30)	<0.001
Age at study (years)	12.21 (7.43; 17.34)	12.68 (9.21; 15.42)	0.905
Follow-up (years)	5.58 (2.25; 8.72)	7.58 (2.01; 9.84)	0.427
ALT (U/L)	17.50 (13.50; 33.00)	13.00 (11.00; 19.00)	0.053
TG (mg/dL)	100.00 (66.00; 139.00)	72.00 (56.00; 98.00)	0.078
E-FABP (ng/mL)	10.86 (9.16; 17.52)	9.78 (7.79; 12.04)	0.055
A-FABP (ng/mL)	27.76 (20.84; 38.74)	20.09 (12.32; 26.21)	0.006

BMI body mass index, ALT alanine aminotransferase, TG triglycerides, E-FABP epidermal fatty acid-binding protein, A-FABP adipocyte fatty acid-binding protein, IQR interquartile range.

**Table 4:** Multivariate analysis of correlates of the fatty acid-binding proteins (FABPs) in acute lymphoblastic leukemia survivors.

	Independent Variable	Coeff.	t	*p*	95% Conf. Interval	
A-FABP (ng/mL)	BMI (kg/m^2^)	1.02	2.87	0.006	0.31	1.73
	SBP (normal vs. high)	13.74	2.04	0.046	0.23	27.3
	DBP (normal vs. high)	−0.64	−0.12	0.907	−11.6	10.3
E-FABP (ng/mL)	BMI (kg/m^2^)	0.48	3.43	0.005	0.17	0.78
	Cholesterol (mg/dL)	0.04	1.40	0.186	−0.02	0.11

A-FABP adipocyte fatty acid-binding protein, E-FABP epidermal fatty acid-binding protein, BMI body mass index, SBP systolic blood pressure, DBP diastolic blood pressure. Coefficient of determination (R^2^) was 0.25 for A-FABP and 0.60 for E-FABP.

**Table 5:** Comparison of childhood cancer survivors with the control group in terms of the number of metabolic derangements.

	≥1 Metabolic Risk Factor	Control Group	*p* Value
	*n* = 33	*n* = 25	
	**Median (IQR)**	**Median (IQR)**	
E-FABP (ng/mL)	11.28 (8.44; 14.60)	10.04 (7.64; 11.68)	0.090
A-FABP (ng/mL)	25.81 (21.32; 37.84)	13.44 (10.48; 21.15)	<0.001
	**≥2 Metabolic Risk Factors**		
	*n* = 10	*n* = 25	
E-FABP (ng/mL)	13.74 (9.88; 16.27)	10.04 (7.64; 11.68)	0.021
A-FABP (ng/mL)	25.51 (24.32; 37.84)	13.44 (10.48; 21.15)	0.001

E-FABP epidermal fatty acid-binding protein, A-FABP adipocyte fatty acid-binding protein, IQR interquartile range.
